# The effects of lactulose on constipation in patients with Parkinson's disease: An exploratory pilot study

**DOI:** 10.1016/j.ensci.2024.100503

**Published:** 2024-05-10

**Authors:** Shin-ichiro Kubo, Mako Ito, Kyoko Matsuba, Tomohiro Shimono

**Affiliations:** a Parkinson's Disease Center, Department of Neurology, Eisei Clinic, 588-17 Kunugidamachi, Hachioji, Tokyo 193-0942, Japan; b Nutrition Department, Eisei Clinic, 588-17 Kunugidamachi, Hachioji, Tokyo 193-0942, Japan; c Morinaga Milk Industry Clinico Co., Ltd., 4-4-22, Meguro, Meguro-Ku, Tokyo 153-0063. Japan

**Keywords:** Constipation, Parkinson's disease, Lactulose, Prebiotics

## Abstract

**Introduction:**

Constipation is one of the most common non-motor symptoms of Parkinson's disease (PD) and is associated with reduced quality of life in patients with PD. The aim of this study was to evaluate the effect of lactulose on defecation status in patients with PD.

**Methods:**

In this open-label, single-center, exploratory pilot study, twenty-nine patients with PD received lactulose for three weeks for the treatment of constipation. The primary endpoint was the number of spontaneous bowel movements (SBMs). The secondary endpoints were stool consistency (Bristol Stool Form Scale [BSFS]) and the number of rescue laxatives used.

**Results:**

Twenty-five patients with PD completed the study. The number of SBMs recorded during the lactulose intervention period was significantly increased compared with that recorded during the pre-intervention period. During the intervention period, the BSFS scores of the patients increased significantly, whereas the number of rescue laxatives they used decreased significantly. No serious adverse events were observed during the study period. Lactulose was well-tolerated.

**Conclusions:**

The results of this study suggest that lactulose may be effective in improving defecation status in patients with PD. Further randomized controlled trials are needed to confirm the effects of lactulose on constipation in patients with PD.

## Highlights

• Constipation is an important determinant of QOL in patients with PD.
• Modulation of gut microbiota has become a potential therapeutic target in PD.
• Lactulose used widely as a laxative has been known to have a prebiotic effect.
• The present study suggests that lactulose may be effective for constipation in PD.
• Further randomized controlled trials are needed to confirm the effects.

## List of Abbreviations

**Table:** 

MMSE	Mini-Mental State Examination
PD	Parkinson's disease
SBM	spontaneous bowel movement

## 1 Introduction

Constipation is one of the most common and distressing non-motor symptoms of Parkinson's disease (PD). Constipation is a potential risk factor for serious complications, such as intestinal pseudo-obstruction, sigmoid volvulus, and acute urinary retention. The exact mechanism underlying constipation in PD is unclear; however, its pathophysiological underpinnings include anorectal dysfunction and slow transit of fecal material through the colon, sometimes in combination. The treatment options for managing constipation in PD are limited and include the use of laxatives; however, only polyethylene glycol and lubiprostone have received evidence-based recommendations as possibly useful treatment options [1].

With the recognition of an association between PD and alterations in gut microbiota [2], modulation of gut microbiota has become a potential therapeutic target in PD [3]. The International Scientific Association for Probiotics and Prebiotics (ISAPP) defines probiotics as “live microorganisms that, when administered in adequate amounts, confer a health benefit on the host”. Similarly, prebiotics are defined as “a substrate that is selectively utilized by host microorganisms conferring a health benefit”. Some studies have demonstrated the effectiveness of fermented milk containing probiotics [4,5] and prebiotic fiber [6] in improving constipation in patients with PD. Lactulose, a synthetic disaccharide composed of galactose and fructose, is also known to have a prebiotic effect, and has been used as an osmotic laxative in the treatment of chronic constipation [7]. However, despite its widespread use, the use of lactulose for the treatment of constipation in patients with PD has not been evaluated in clinical trials. Thus, the aim of this study was to evaluate the effect of lactulose on defecation status in patients with PD.

## 2 Methods

### 2.1 Patients and study design

In this open-label, single-center, exploratory pilot study, twenty-nine patients with PD who visited the PD Center of Eisei Clinic and reported the presence of purely subjective constipation were consecutively recruited. All the patients fulfilled the UK Parkinson's Disease Society Brain Bank criteria for idiopathic PD. The exclusion criteria were as follows: cognitive decline (Mini-Mental State Examination (MMSE) score < 21); receiving device-aided therapies, such as levodopa/carbidopa intestinal gel and deep brain stimulation; and comorbid conditions that prevent reliable completion of study assessments. Dopaminergic treatments were unchanged during this study period.

The study period consisted of a three-week pre-intervention period and a three-week intervention period. During the intervention period, the patients consumed 15 mL of lactulose syrup (10 g lactulose/15 mL) per day. Up to 45 mL per day was allowed depending on the patient's defecation status. Regular use of laxatives was continued and the use of rescue laxatives was not restricted. During the study period, patients used a defecation diary to record the following variables: the number of bowel movements; stool consistency (scored using the 7-point Bristol Stool Form Scale [1 = hard lumps, 2 = lumpy sausage, 3 = cracked sausage, 4 = smooth sausage, 5 = soft lumps, 6 = mushy, 7 = watery]) [8]; and the use of rescue laxatives.

The primary endpoint was the number of spontaneous bowel movements (SBM). SBM was defined as occurrence of a bowel movement within 24 h without using a rescue laxative. The secondary endpoints were stool consistency (Bristol Stool Form Scale) of SBMs and the number of rescue laxatives used.

The study was approved by the ethics committee of Eisei Clinic and conducted in compliance with the principles of the Declaration of Helsinki. All the participants provided written informed consent.

### 2.2 Statistical analyses

Data were analyzed using SPSS software (IBM SPSS Statistics for Windows, Version 24.0. Armonk, NY: IBM Corp.). All variables were tested for normality using the Shapiro-Wilk test. If variables were normally distributed, the differences between the pre-intervention and intervention values were compared using paired *t*-tests. If at least one variable (pre-intervention or intervention) was not normally distributed, the differences between the pre-intervention and intervention values were compared using the Wilcoxon signed-rank test. *P* < 0.05 was considered statistically significant.

## 3 Results

Of the 29 patients enrolled in this study, 25 patients (12 males; age 75.4 ± 6.3 years) completed the study. Four patients discontinued the study for different reasons (syrup was too sweet to consume, *n* = 1; unable to complete stool diary, n = 1; adverse events, *n* = 2). Regarding clinical characteristics, the average duration of PD was 6.9 ± 5.0 years, Hoehn and Yahr scale score was 2.7 ± 0.6, MMSE score was 27.4 ± 2.4, levodopa-equivalent dosage was 550 ± 304 mg per day, and lactulose intake was 12.6 ± 5.2 g per day.

**Fig. 1:**
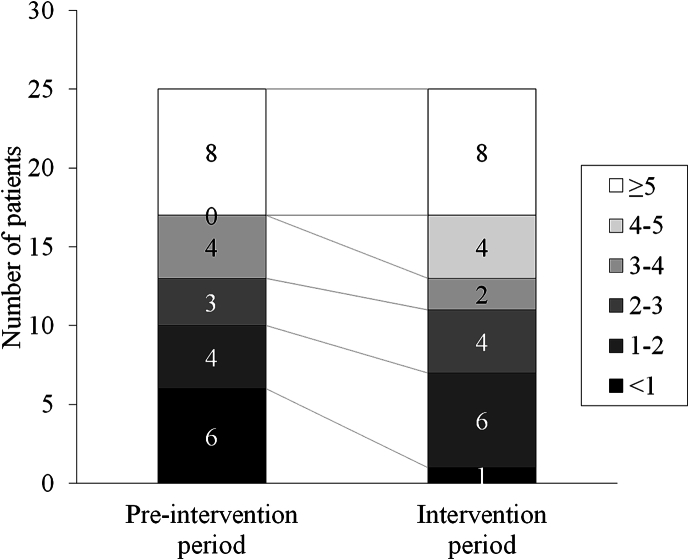
The number of patients for each spontaneous bowel movements. Each square represents the number of spontaneous bowel movements per week.

Regarding the primary endpoint, the number of SBMs recorded during the intervention period was significantly higher than that recorded during the pre-intervention period. For the secondary endpoint, stool consistency (Bristol Stool Form Scale) increased significantly and the number of rescue laxatives used decreased significantly during the intervention period (Table 1 and Fig. 1).

**Table 1:** The effects of lactulose on defecation status in patients with Parkinson's disease.

	Pre-intervention period	Intervention period	*P*-value
Number of SBMs (per week) 1	3.08 ± 2.48	3.79 ± 2.40	0.011
Bristol Stool Form Scale score^1^	3.22 ± 1.22	3.66 ± 1.28	0.005
Number of rescue laxatives used (per three weeks)^2^	0 [0, 5]	0 [0,3]	0.046

SBM, spontaneous bowel movement.

1 Values are expressed as mean ± SD. P values were calculated using paired t-tests.

2 Values are expressed as median [25th and 75th percentiles]. *P* values were calculated using Wilcoxon signed-rank test.

Two adverse events (worsening of stool characteristics) were reported. No serious adverse events were observed during the study period.

## 4 Discussion

The results of this study suggest that lactulose may increase the number of SBMs in patients with PD who have subjective constipation. In addition, the results showed that lactulose may improve stool consistency and reduce the use of rescue laxatives. These results are consistent with those of previous studies on the modulation of gut microbiota in patients with PD who have constipation [[4], [5], [6]]. In the first pilot study on the effects of milk fermented using the probiotic strain *Lactobacillus casei* Shirota, the treated patients showed significant improvements in stool consistency and bowel habits [4]. Since then, the effects of prebiotic fiber in addition to fermented milk containing multiple probiotic strains on constipation associated with PD has been demonstrated [6]. Although lactulose has been used empirically in the treatment of constipation in patients with PD, to the best of our knowledge, the present study is the first in which the effects of “prebiotic” lactulose as the sole therapy for constipation in patients with PD were evaluated. Intriguingly, in a randomized placebo-controlled study of probiotics for the treatment of constipation in PD, 32.4% of patients in the active treatment group were using lactulose at baseline [5].

Evidence of alterations in gut microbiota provided by multiple case-control studies conducted using diverse populations suggest that bowel dysfunction in PD may occur alongside dysbiosis [9]. However, whether the previously reported changes in gut microbiota play a causal role in the development of gut pathology in PD or whether they are a consequence of altered gut function remain unclear. Nevertheless, observations that the PD phenotype, including gut dysmotility and inflammation, in alpha-synuclein-overexpressing transgenic mice can be modulated by manipulating their gut microbiota suggest that such causal effects are possible [10].

Lactulose is commonly used in many countries as a functional food that promotes intestinal transit. As lactulose is not digested by digestive enzymes, it reaches the colon directly without being absorbed in the stomach or small intestine and exerts its effects by promoting the growth of health-promoting intestinal bacteria and the production of beneficial metabolites, such as short-chain fatty acids. Therefore, lactulose may be effective for treating constipation and digestive disorders, such as inflammatory bowel disease [11]. Given that alterations in gut microbiota, permeability, short-chain fatty acids, and gut inflammation are reported to be associated with age at the onset of PD [12], it is reasonable to envisage that lactulose may play a role in the future therapeutic landscape of PD in terms of disease-modifying treatments, based on the concept of a microbiota-gut-brain axis [2,3]. However, changes in gut microbiota were not examined in the present study; thus, its effects on the pathogenesis of PD remain unknown. Future studies should be focused on gut microbiota, its metabolites, and the assessment of inflammation.

This study had some limitations. First, the number of patients enrolled in this study was small. Second, placebo controls were not included for comparison, and there was no “wash-out period” following the intervention period, which could have provided more data on potentially waning effects. Third, this was a single-center study. Finally, the use of laxatives was not restricted, and overall diet and daily fluid intake were not controlled; therefore, the combined effect of lactulose and laxatives, as well as that of lactulose and food or beverage intake, cannot be completely excluded.

In conclusion, lactulose may have beneficial effects in the treatment of patients with PD and subjective constipation. The results of this study may be foundational for future randomized, double-blind, placebo-controlled studies and investigations on the effect of lactulose on the pathogenesis of PD.

## Ethics approval and consent to participate

This study was approved by the institutional ethics committee of Eisei Hospital (approval number: 2022–003).

## Consent for publication

Written consent to publish was obtained from the patients.

## Availability of data and materials

Data that support the findings presented in this study are available from the corresponding author upon reasonable request.

## Funding

This report did not receive any specific grants from funding agencies in the public, commercial, or not-for-profit sectors.

## CRediT authorship contribution statement

**Shin-ichiro Kubo:** Conceptualization, Data curation, Methodology, Project administration, Supervision, Writing – original draft, Writing – review & editing. **Mako Ito:** Data curation, Methodology, Writing – review & editing. **Kyoko Matsuba:** Data curation, Methodology, Writing – review & editing. **Tomohiro Shimono:** Conceptualization, Data curation, Methodology, Resources, Software, Writing – review & editing.

## Declaration of competing interest

The authors declare that they have no competing interests.
